# Cascade of zero-field Chern insulators in magic-angle bilayer graphene

**DOI:** 10.1093/nsr/nwaf265

**Published:** 2025-07-03

**Authors:** Zaizhe Zhang, Jingxin Yang, Bo Xie, Zuo Feng, Shu Zhang, Kenji Watanabe, Takashi Taniguchi, Xiaoxia Yang, Qing Dai, Donghua Liu, Kaihui Liu, Zhida Song, Tao Liu, Jianpeng Liu, Xiaobo Lu

**Affiliations:** International Center for Quantum Materials, School of Physics, Peking University, Beijing 100871, China; International Center for Quantum Materials, School of Physics, Peking University, Beijing 100871, China; National Engineering Research Center of Electromagnetic Radiation Control Materials, School of Electronic Science and Engineering, University of Electronic Science and Technology of China, Chengdu 611731, China; School of Physical Science and Technology, ShanghaiTech University, Shanghai 201210, China; State Key Laboratory for Mesoscopic Physics, Frontiers Science Centre for Nano-optoelectronics, School of Physics, Peking University, Beijing 100871, China; CAS Key Laboratory of Nanophotonic Materials and Devices, CAS Key Laboratory of Standardization and Measurement for Nanotechnology, CAS Center for Excellence in Nanoscience, National Center for Nanoscience and Technology, Beijing 100871, China; Research Center for Electronic and Optical Materials, National Institute of Material Sciences, Tsukuba 305-0044, Japan; Research Center for Materials Nanoarchitectonics, National Institute of Material Sciences, Tsukuba 305-0044, Japan; CAS Key Laboratory of Nanophotonic Materials and Devices, CAS Key Laboratory of Standardization and Measurement for Nanotechnology, CAS Center for Excellence in Nanoscience, National Center for Nanoscience and Technology, Beijing 100871, China; CAS Key Laboratory of Nanophotonic Materials and Devices, CAS Key Laboratory of Standardization and Measurement for Nanotechnology, CAS Center for Excellence in Nanoscience, National Center for Nanoscience and Technology, Beijing 100871, China; School of Materials Science and Engineering, Shanghai Jiao Tong University, Shanghai 200240, China; School of Materials and Energy, University of Electronic Science and Technology of China, Chengdu 611731, China; State Key Laboratory for Mesoscopic Physics, Frontiers Science Centre for Nano-optoelectronics, School of Physics, Peking University, Beijing 100871, China; International Center for Quantum Materials, School of Physics, Peking University, Beijing 100871, China; Collaborative Innovation Center of Quantum Matter, Beijing 100871, China; Hefei National Laboratory, Hefei 230088, China; National Engineering Research Center of Electromagnetic Radiation Control Materials, School of Electronic Science and Engineering, University of Electronic Science and Technology of China, Chengdu 611731, China; School of Physical Science and Technology, ShanghaiTech University, Shanghai 201210, China; International Center for Quantum Materials, School of Physics, Peking University, Beijing 100871, China; Collaborative Innovation Center of Quantum Matter, Beijing 100871, China

**Keywords:** magic angle graphene, Chern insulators, topological flat band

## Abstract

The interplay between strong electron-electron interactions and symmetry breaking can have a profound influence on the topological properties of materials. In magic-angle twisted bilayer graphene (MATBG), the flat band with a single SU(4) flavor associated with the spin and valley degrees of freedom gains a non-zero Chern number when *C*_2*z*_ symmetry or *C*_2*z*_*T* symmetry is broken. Electron-electron interactions can further lift the SU(4) degeneracy, leading to Chern insulator states. Here, we report a complete sequence of zero-field Chern insulators at all odd-integer fillings (*ν* = ±1, ±3) with different chiralities (*C* = 1 or −1) in hBN-aligned MATBG which structurally breaks *C*_2*z*_ symmetry. The Chern states at hole fillings (*v* = −1, −3), which are firstly observed in this work, host an opposite chirality compared with the electron filling scenario. Furthermore, at the valence band filling *ν* = −7/2, the zero-field symmetry broken Chern insulator with *C* = −1 can be observed. Remarkably, a prominent Streda-formula violation around the *v* = −3 state has been observed. By doping the Chern gap at *v* = −3 with a notable number of electrons at finite magnetic field, the Hall resistance *R*_yx_ robustly quantizes to ∼*h*/*e*^2^, whereas the longitudinal resistance *R*_xx_ vanishes, indicating that the chemical potential is pinned within a Chern gap, forming the magnetic field stabilized incommensurate Chern insulator states. By providing the first experimental observation of zero-field Chern insulators in the flat valence band, our work fills up the overall topological framework of MATBG with broken *C*_2*z*_ symmetry.

## Teaser

This study reveals multiple zero-field Chern insulators and emergent correlated quantum crystal phases in aligned magic-angle bilayer graphene.

## INTRODUCTION

Flat band in magic-angle twisted bilayer graphene (MATBG) offers an ideal platform [1] to study novel band topology as well as other quantum phases including superconductivity [2–7], correlated insulators [8] and orbital magnetism [9–20], etc. [21–26]. From a single-particle perspective, the lowest flat valence and conduction bands of MATBG touch at Dirac points, which are protected by *C*_2*z*_*T* symmetry. Breaking either *C*_2*z*_ symmetry or *C*_2*z*_*T* symmetry can gap out the Dirac point and lead to a net Chern number of *C* = 1 or *C* = −1 for each flat band within a single SU(4) isospin flavor [27–37]. Past studies [10–12] have shown that the *C*_2*z*_ symmetry of graphene can be effectively broken by making lattice alignment with hexagonal boron nitride (hBN) and Chern insulator at zero magnetic field has been observed with moiré filling *ν* = +3 (three electrons filled in each moiré unit cell). Later studies [15,20] have shown that the strong electron correlations in MATBG could also break the *C*_2*z*_*T* symmetry even without alignment to hBN substrates, giving rise to different Chern insulator states, including

*v* = +1 (one electron filled in each moiré unit cell) state with a Chern number of *C* = −1 which is robust down to zero magnetic field as inferred from Streda formula behavior of *R*_xx_ minima, and a sequence of Chern insulators onset under finite magnetic fields.

However, so far Chern insulator states at zero magnetic field characterized by the (quantized) anomalous Hall effect in MATBG have only been observed in the conduction band, and the quantized *R*_yx_ has been only observed at filling *ν* = +3. The topological nature of the valence flat band remains not well understood. Theoretically, the exact topological properties of the correlated states in MATBG highly rely on the interlayer hopping parameters, i.e. *w*_0_ for AA interlayer hopping and *w*_1_ for AB interlayer hopping [35], which are also closely related to other quantum phases including superconductivity and magnetism in this system [38]. Experimentally mapping out the overall topological framework in particular within a single MATBG device will be both inspirational and informative, but also challenging due to the difficulty of controlling twist angles and the fragility of these Chern insulator states.

**Figure 1.**
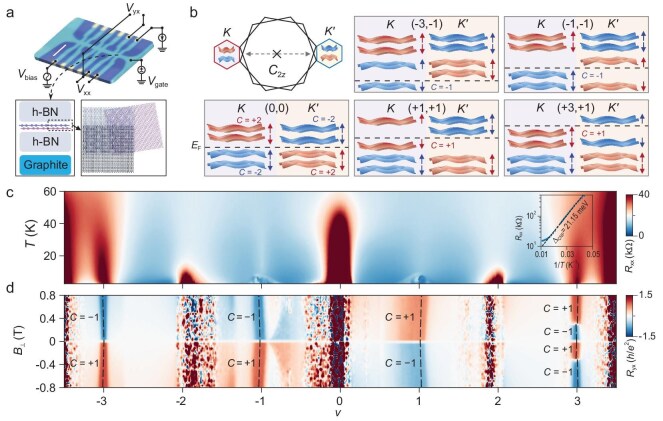
Chern insulators in MATBG aligned to hBN. (a) Optical microscopy image (scale bar is 5$\mu $m) of device D1 and measurement configuration. Four terminal transport measurements for longitudinal resistance *R*_xx_ and Hall resistance *R*_yx_ are achieved by applying an alternating excitation *V*_bias_ and measuring the voltage drop *V*_xx_, *V*_yx_ and the current *I* flowing through the device (top panel). The cross-section and schematic display that the MATBG with hBN encapsulation is further aligned with top hBN and gated with graphite (bottom panel). (b) Mini-Brillouin zone of MATBG with *C*_2*z*_ symmetry breaking and different Chern band configurations with (*v, C*) indices of (0, 0), (−3, −1), (−1, −1), (+1, +1) and (+3, +1). (c) Temperature dependence of *R*_xx_ (measured in a cryogenic refrigerator with a base temperature of 2.7 K) in device D1, the illustration in the upper right corner shows the Arrhenius plot for *v* = 0 (CNP), with an extracted gap of △_CNP_ = 21.15 meV. The activation energy gap at the CNP is very large, strongly suggesting a perfect alignment between MATBG and the hBN substrate. (d) Anti-symmetrized (see Methods for details) *R*_yx_ versus filling factor *v* and out-of-plane magnetic field *B*_⊥_ obtained at *T* = 10 mK in device D1. The slanted dashed lines in the figure represent the evolution of the Chern insulator states (−3, −1), (−1, −1), (+1, +1) and (+3, +1) with the magnetic field according to the Streda formula.

In this work, we report the observation of multiple commensurate Chern insulator states with opposite chiralities on the electron and hole sides, as well as the symmetry broken Chern insulator at *v* = −7/2 in MATBG aligned to an hBN substrate (Fig. 1a).

## RESULTS

### Complete sequence of Chern insulators

As shown in Fig. 1b, the staggered sublattice potential induced by the aligned hBN substrate can effectively break the *C*_2*z*_ symmetry, leading to the emergence of opposite valley Chern numbers for the flat bands. At zero magnetic field, the Chern insulator states can be spontaneously polarized into either valley driven by interactions, since the flat bands of the two opposite valleys are energetically degenerate at the non-interacting level. Given the same valley polarization, Fig. 1b shows the filling configurations of the flat band with single isospin flavor for different Chern states observed in our experiment. Figure 1c shows *R*_xx_ of device D1 (twist angle *θ* ∼1.15°) as a function of temperature *T* and moiré filling factor *v*. Similar to previously reported results, resistive correlated states at integer moiré fillings can be resolved [2–4,8]. As a result of substrate induced *C*_2*z*_ symmetry breaking, the charge neutrality point (CNP) exhibits a sizable gap (Δ_CNP_ ≈ 21.15 meV) with clear thermal activation shown in the inset of Fig. 1c. Figure 1d shows anti-symmetrized Hall resistance *R*_yx_ as a function of filling factor *v* (fast axis and sweeping from left to right) and perpendicular magnetic field *B*_⊥_ measured at a based phonon temperature of *T* ∼10 mK. When the carrier density is close to values associated with odd integer fillings (*v* = ±1, ±3), *R*_yx_ undergoes an abrupt sign reversal at zero magnetic field which is further illustrated in Fig. S1 and highly implying the formation of zero-field Chern insulators. In the conduction band, *R*_yx_ values at *v* = 1 and *v* = 3 can reach ∼70% and 95% of one quantized resistance value (*h*/*e*^2^) at *B*_⊥_ = 480 mT (Fig. S1(e)), respectively, which is consistent with previous work. Interestingly, we first observe zero-field Chern insulators at the flat valence band of MATBG with Hall resistance reaching 98% for the *v* = −3 state and 89% for the *v* = −1 state at *B*_⊥_ = 480 mT. As clearly shown in Fig. 1d, the Chern insulator for *v* = −3 (*v* = −1) shows an opposite Chern number with the *v* = 3 (*v* = 1) state (when *B*_⊥_ >300 mT). These experimental results are in good agreement with our Hartree–Fock calculations (see Methods and Fig. S2), which further confirm the topological nature of the ground states in MATBG with *C*_2*z*_ symmetry breaking. To summarize briefly, the magnetization of MATBG which is dominated by orbital magnetism is energetically favored to align with the external *B*_⊥_ field. At finite *B*_⊥_ field, as shown in Fig. 1b, the Chern insulator states at integer hole filling and integer electron filling are polarized to the same valley (same orbital magnetization) but with opposite Chern numbers.

It is noteworthy that in Fig. 1c there is additional sign reversal of *R*_yx_ for the *v* = 3 state at *B*_⊥_ ≈ ±300 mT. Careful measurements show that the sign change of *R*_yx_ sensitively depends on the sweeping direction of *v*. As shown in Fig. S3 which is measured with opposite sweeping direction of *v* (from right to left), the sign reversal behavior of *R*_yx_ appears at *v* = −3. Similar sign reversal of *R*_yx_ has also been reported at the *v* = 1 state and other systems showing Chern insulators [20,39–43] and can be attributed to the competition between the two components of orbital magnetization including Chern magnetization contributed by the gapless edge states and magnetization from self-rotation of the wavepacket [20,44]. We also note that our device does not exhibit zero-field Chern insulators at *v* = ±2, indicating the absence of valley polarization for *v* = ±2 states [41]. Furthermore, topologically trivial charge density wave (CDW) states can be resolved at fractional moiré fillings (Fig. S1(c) and (d)). The *v* = −2/3 state starts to appear at *B*_⊥_ = ±0.5 T whereas the *v* = 5/3 state can be stabilized at *B*_⊥_ = ±0.2 T.

**Figure 2.**
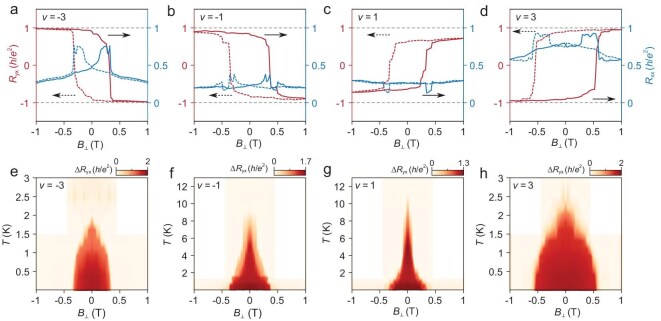
Magnetic hysteresis loops. (a–d) Symmetrized *R*_xx_ and anti-symmetrized *R*_yx_ as a function of *B*_⊥_ measured at *ν* = ±1 and ±3 states, *T* = 10 mK. Dashed and solid lines correspond to sweeping the *B*_⊥_ field back and forth indicated by the arrows. (e–h) Δ*R*_yx_ = |*R*_yx_ (*B*_⊥_ sweeping up)−*R*_yx_ (*B*_⊥_ sweeping down)| versus *B*_⊥_ field at different temperatures measured at *ν* = ±1 and ±3 states.

To further investigate the property of Chern insulators, we measured the magnetic response for these states with fixed carrier density. In Fig. 2a–d, we display the hysteresis loops of *R*_yx_ and *R*_xx_ as the magnetic field is swept back and forth at different moiré fillings, showing Chern insulators with *R*_yx_ values at zero magnetic field reaching 90.2% (*v* = −3), 83.5% (*v* = −1), 63.7% (*v* = 1) and 91.3% (*v* = 3). The imperfect quantization of *R*_yx_ and non-zero *R*_xx_ is due to non-uniform magnetic domains and concomitant percolation in the Chern insulator [20]. We note that the additional sign change of *v* = ±3 states is missing in the magnetic hysteresis loop wherein the chemical potential and magnitude of the magnetization are fixed. Notably, the initially observed Chern insulators at *v* = −3 and *v* = −1 states exhibit magnetic hysteresis loops with chirality exactly opposite to those in *v* = 3 and *v* = 1 states, which is consistent with our Hartree–Fock calculation showing opposite Chern numbers between the Chern insulators with *v* >0 and *v* <0, given the same valley polarization (Fig. S2).

Figure 2e–h illustrates the temperature dependence of magnetic hysteresis loops measured at *v* = ±1 and *v* = ±3 states in device D1. Both *v* = ±3 states exhibit comparable Curie temperatures which are defined by the point where the magnetic hysteresis loops of *R*_yx_ vanish. As shown in Fig. 2e and h, the Curie temperatures are ∼*T*_c_ = 2.0 K for *v* = −3 and *T*_c_ = 2.4 K for *v* = 3. Interestingly, the *v* = +1 state with *T*_c_ = 10.7 K and *v* = −1 state with *T*_c_ = 8.6 K have notably higher Curie temperatures than *v* = ±3 states. Whereas the *v* = ±1 state exhibits less perfect quantization of *R*_yx_. Similar behaviors have also been observed in the measurement of *R*_xx_ as a function of temperature *T* and filling factor *ν* under zero *B*_⊥_ field (Fig. S6). The abrupt change of *R*_xx_ with decreasing temperature indicates the emergence of orbital magnetism along with the formation of Chern gaps. All these features have been reproduced in different contact pairs of device D1 (Fig. S4) and device D2 (Fig. S5). Moreover, device D2 exhibits even better zero-field quantization of the Hall resistance at *v* = +1 and −3 after a further reduction of the functional region in order to improve structural homogeneity (Fig. S9).

### Symmetry broken Chern insulator at *v* = −7/2

**Figure 3.**
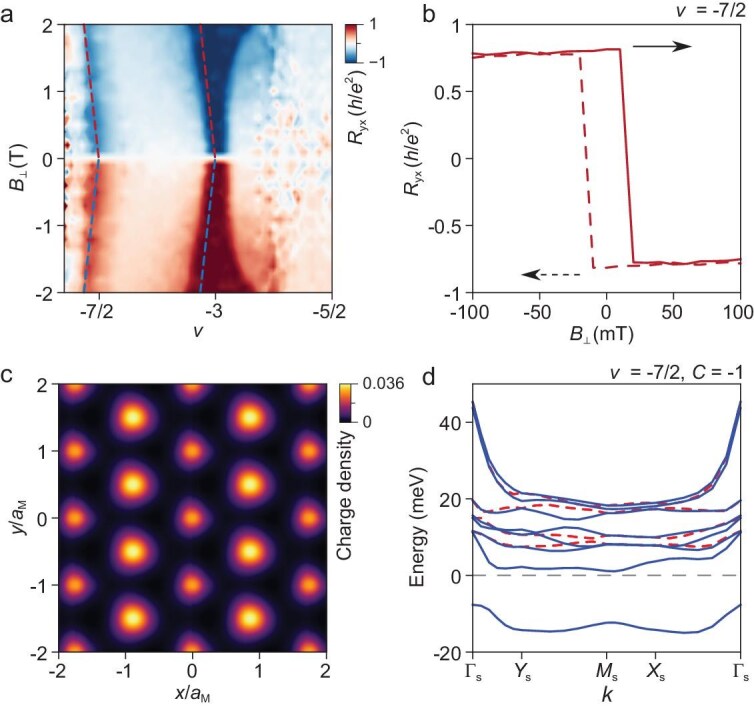
Symmetry broken Chern insulator at *v* = −7/2 in device D2. (a) Anti-symmetrized *R*_yx_ as a function of magnetic field *B*_⊥_ and moiré filling factor *v*. The dashed lines in the figure represent the evolution of the Chern insulator state (−7/2, −1) and (−3, −1) with the magnetic field according to the Streda formula after reducing the sample area (see Fig. S9 for more details). (b) Hysteresis loop measured at *v* = −7/2. Dashed and solid lines correspond to sweeping the out-of-plane magnetic field *B*_⊥_ in opposite directions, as indicated by the arrows. (a and b) employ the measurement configuration shown in Fig. S9(e). (c) Electron charge density distribution calculated for the (−7/2, −1) SBCI state, where the *a*_M_ represents the effective periodicity of the moiré superlattice. (d) Band structure of the MATBG at the filling factor of *ν* = −7/2. The red dashed lines and the blue lines denote the energy bands from two atomic valleys and the gray dashed line indicates the Fermi level. The Chern number of the occupied bands is *C* = −1 for *ν* = −7/2 under zero magnetic field. All experimental data are acquired at *T* = 10 mK.

Compared to device D1, device D2 has a global bottom gate which can effectively reduce the structural inhomogeneity (such as gating PN junctions) near the leads. In this case, better resolution close to the band insulator at *v* = −4 can be achieved. Interestingly, we have observed clear signatures of a Chern insulator at *ν* = −7/2. Figure 3a shows the anti-symmetrized *R*_yx_ as a function of magnetic field *B*_⊥_ and moiré filling factor *v*. The evolution of the state at *ν* = −7/2 shows clear magnetic field dependence, with the trajectory indicating a Chern insulator (*C* = −1). Figure 3b shows the hysteresis loop of the significant anomalous Hall resistance *R*_yx_ at *ν* = −7/2. Similar with the Chern insulators at *ν* = ±1 and ±3, even with an applied out-of-plane magnetic field, this Hall resistance does not reach a quantized Hall resistance of *h/e*^2^ due to the disorder (Fig. S9). The integer Chern insulator at fractional filling with anomalous Hall effect has been observed in twisted multilayer graphene [45,46], which was first reported in MATBG. To further understand the ground state of such a Chern insulator, we have performed Hartree–Fock calculations at *v* = −7/2 (Fig. 3c and d) which show spontaneous breaking of the translation symmetry. At *v* = −7/2, the system exhibits a stripe phase, accompanied by unit-cell doubling which will further fold the moiré bands. The new folded sub-moiré band carries a non-zero Chern number of *C* = ±1. At *ν* = −7/2, electrons occupy only the lowest sub-moiré band with *C* = −1, which is consistent with our experimental observations.

### Magnetic field stabilized incommensurate Chern insulators near *v* = −3

**Figure 4.**
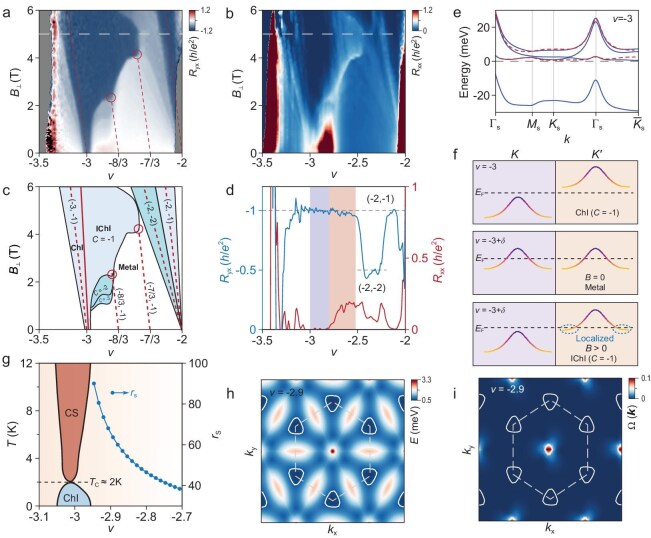
Magnetic field stabilized IChI states near *v* = −3. (a and b) Unsymmetrized Landau fan diagram of the *R*_yx_ (anti-symmetrized *R*_yx_ is shown in Fig. S7) and *R*_xx_. The red dashed lines represent the evolution of Chern insulator states with Chern number *C* = −1 emanating from *ν* = −3, −8/3, −7/3, and −2 based on the Streda formula at *T* = 10 mK. (c) Schematic diagram indicating the Chern insulator states with (*v, C*) indices of (−3, −1), (−8/3, −1), (−7/3, −1), (−2, −2) and (−2, −1) extracted from the Landau fan diagram (a). (d) Line cuts of *R*_xx_ and *R*_yx_ at *B*_⊥_ = 5 T (corresponding to the gray dashed lines in (a) and (b)). The purple region indicates where *R*_xx_ vanishes completely to zero, while the orange region signifies where *R*_xx_ begins to exhibit non-zero values. In both regions, *R*_yx_ shows quantized values of −*h*/*e*^2^. (e) Band structure of the MATBG at the filling factor of *ν* = −3. The red dashed lines and the blue lines denote the energy bands from two atomic valleys and the gray dashed line indicates the Fermi level. (f) Schematic illustration of ground states of ChI, metal and IChI, the colors in the top and bottom figures qualitatively represent the distribution of the Berry curvature; the Berry curvature gradually increases from yellow to purple. (g) Schematic diagram illustrating the different correlated phases near *ν* = −3, extracted from Fig. S6(b) and the Wigner–Seitz radius *r*_s_ obtained from theoretical calculations, as a function of the filling factor *ν* (right y-axis), where CS denotes the resistive correlated state and ChI denotes the Chern insulator. (h) Band structure of the unoccupied flat band above the *v* = −3 gap and Fermi surface when the filling factor is *ν* = −2.9. The gray dashed lines show the moiré Brillouin zone and the white lines represent the Fermi surface. (i) Distribution of the Berry curvature in the unoccupied flat band above the *v* = −3 gap. The Chern number of the unoccupied band is *C* = +1. The hot spots of the Berry curvature are localized near the *Γ*_s_ point, while becoming very small near *K*_s_ and *M*_s_ points. The gray dashed lines also show the moiré Brillouin zone, while the white lines represent the same Fermi surface as shown in (h).

Exotic phenomena have been observed in the *B*_⊥_ dependent behavior of the Chern insulator (ChI) near *v* = −3. Generally, the Hall resistance *R*_yx_ changes its sign at finite *B*_⊥_ field when the chemical potential is tuned across a topologically trivial gap. For a Chern gap scenario, the evolution of the gap in *n*-*B*_⊥_ (*n* is the carrier density) parameter space is further expected to follow the Streda formula $\frac{{{\mathrm{d}}n}}{{{\mathrm{d}}{B_ \bot }}} = \frac{{Ce}}{h}$ where *C* is the Chern number, *e* is the electron charge and *h* is Planck's constant. Strikingly, the behavior of the *v* = −3 state violates the Streda formula, with neither a sign reversal of *R*_yx_ at *v* = −3 nor a standard trajectory following the slope of $\frac{{{\mathrm{d}}n}}{{{\mathrm{d}}{B_ \bot }}} = - \frac{e}{h}$ has been observed. Instead, the *v* = −3 state persists over a very broad region (rendered in dark blue in Fig. 4a) with *C* = −1 in the *v*-*B*_⊥_ parameter space (Fig. 4a and b). Figure 4d further quantitatively shows the quantized *R*_yx_ and vanishing *R*_xx_ at *B*_⊥_ = 5 T for the Chern gap stemming from *v* = −3 and the conventional Landau level (LL) gaps from *v* = −2. In Fig. 4c, the schematic shows the broad *C* = −1 state at *v* = −3 as well as the different LL gaps fanning out from *v* = −2. For the *C* = −1 state at *v* = −3, the red solid line qualitatively indicates an imaginary boundary between the normal Chern gap (leftward) which follows the Streda formula and electron doped Chern gap (rightward) which robustly shows quantized *R*_yx_ but violates the Streda formula (referred to as Incommensurate Chern Insulators, IChI). We note that the significant broadening feature is absent for the Landau level gaps originated from *v* = −3 and −2, which can exclude the dominant origin of disorder effect. Furthermore, the two corners indicated by red circles in Fig. 4a can be accurately linked to *v* = −8/3 and −7/3 at zero *B*_⊥_ field with a trajectory corresponding to *C* = −1, indicating the enhancement of topological states at commensurate fractional moiré fillings, *v* = −8/3 and −7/3. The absence of a phase boundary between the commensurate states and incommensurate states demonstrates that the additionally doped electrons spontaneously break the translational symmetry and condense into localized bulk states (which will be elaborated on later). As shown in Fig. 4d, with more electrons doped (orange region), the localized states start to contribute conductivity. One possible reason for this is that the localized states start to melt with more electrons doped.

In addition, similar states with prominent Streda-formula violations have been observed proximate to the (*v, C*) = (−8/3, −1) state, showing *C* = −2, −3, etc. (Fig. S7). These states stabilized at a finite field can be attributed to the Landau level excitations from *v* = −8/3.

The topologically trivial state at *v* = −2 indicates its valley-unpolarized ground state. Two flat valence bands from different valleys with opposite valley Chern numbers are filled at *v* = −2, leading to a net Chern number of zero. For the ChI at *v* = −3, only a single-flavor Chern band is filled as shown in the top panel of Fig. 4f with quantitative band structure shown in Fig. 4e (more details are shown in Methods). Figure 4f qualitatively shows different microscopic pictures for the different quantum phases shown in Fig. 4c. When the *v* = −3 state is doped with more electrons, all electrons will relocate equally into both valleys, forming a valley balanced metal (middle panel of Fig. 4f) which is evidenced by vanishing anomalous Hall resistance. As *B*_⊥_ field energetically favors both spin and valley polarization, the zero-field metallic state will tend to transition into a valley-polarized state with an external *B*_⊥_ field, leading to a fully filled flavor at the *K* valley and slightly filled flavor at the *K′* valley (bottom panel of Fig. 4f). Figure 4g shows the calculated filling-dependent Wigner–Seitz radius *r*_s_ = m*e^2^/4π${\hbar}$^2^*ε*_0_*ε*_r_(π*n*)^1/2^ which characterizes the ratio of Coulomb repulsion to kinetic energy [47–49]. Here, m*, e, *n*, ${\hbar}$, *ε*_0_ and *ε*_r_ denote the effective electron mass, elementary charge, carrier density, reduced Planck constant, vacuum and relative permittivity, respectively. The electrons in the *K′* valley as illustrated in the bottom panel of Fig. 4f will be localized due to the large *r*_s_ values originating from large effective mass [50–56] and low carrier density.

Figure 4h and i show the calculated distribution of electron pockets of the slightly filled *K′* valley at *v* = −2.9 on top of energy dispersion and Berry curvature distribution in the momentum space, respectively (see Methods for details). Interestingly, these electron pockets have vanishing Berry curvature, as Berry curvature is mainly concentrated at the *Γ*_s_ point (Fig. 4i). This gives rise to the IChI state which maintains the topological property of the *v* = −3 state (bottom panel of Fig. 4f).

A similar extended Chern insulator has been observed in pentalayer rhombohedral graphene at zero magnetic field. Such an incommensurate Chern insulator is reminiscent of the re-entrant quantum Hall states which host Wigner crystals around integer fillings of Landau levels at high magnetic field [57–66]. However, we cannot rule out other possible ground states for the localized bulk states, such as quantum anomalous Hall crystals recently proposed [46,67–75] and incommensurate charge density waves [76–78]. All possible pictures for the extended Chern insulators observed in our experiment need a large *r*_s_ value whereas the exact nature of the state calls for future studies.

## DISCUSSION

Our work for the first time demonstrates the full sequence of zero-field Chern insulators at all odd integer fillings (*ν* = ±1, ±3) and symmetry broken Chern insulators at valence band filling (*ν* = −7/2) which are theoretically predicted in MATBG aligned to hBN. At incommensurate fillings, we have observed magnetic field stabilized topologically nontrivial incommensurate Chern insulator states extended over a broad region in the *ν*-*B*_⊥_ space, by virtue of ultra-large *r*_s_ values. We speculate that the partially valley polarized IChI can also exist in other systems, i.e. rhombohedral multilayer graphene moiré superlattices [79–81], when the *r*_s_ values are large enough. Additional insight into the IChI state can be obtained from frequency-dependent transport measurement, which can reveal its dynamic behavior. Another promising extension of our work would be scanning probe microscope measurement of the system to show the smoking-gun evidence for possible Wigner crystallization.

## METHODS

### Device fabrication

Device D1 was fabricated using a standard ‘cut-and-stack’ dry transfer method. Graphene, graphite and hBN flakes were exfoliated on O_2_ plasma cleaned (30 W, 2 mins) Si^++^/SiO_2_ (285 nm) chips. The graphene flake was cut into several pieces by a femtosecond laser (central wavelength ∼517 nm, pulse width ∼150 fs, repetition frequency ∼80 MHz and maximum power ∼150 mW). A PC (poly bisphenol A carbonate)/PDMS (polydimethylsiloxane) stamp was used to pick up the top hBN, two pieces of graphene, bottom hBN and back gate, sequentially. The crystalline edges of the graphene and hBN flakes were meticulously aligned during the transfer process (Fig. S8(a)). The whole heterostructure was then released onto a Si^++^/SiO_2_ substrate when the PC was backed to 180°C. Finally, the device was defined into the Hall bar geometry utilizing standard electron beam lithography (EBL) and reactive ion etching (RIE) techniques, and the edge contacts (5 nm Cr/70 nm Au) were formed using a combination of electron beam evaporation and thermal evaporation techniques.

For device D2, we adopted a different stacking method to that of D1. The top hBN was first picked up by a PPC (polypropylene carbonate)/PDMS stamp at 50°C and then released onto a graphene flake, with the crystalline edges of graphene and hBN flakes aligned (Fig. S8(b) and (c)). We increased the temperature to 60°C to release the stamp with the PPC film peeled off from hBN, leaving the hBN flake on top of the graphene flake. A laser was then employed to precisely cut off the graphene along the boundary of the top hBN to ensure that the edges of both flakes can be locked to each other. A PC/PDMS stamp was used to pick up the top hBN and underlying graphene, subsequently rotating and transferring them onto the remaining graphene. Trichloromethane (CHCl_3_) was utilized to dissolve the PC on the sample, and then repeating the previous step of using a laser to cut off the graphene along the edges of the hBN. Finally, the top hBN and two pieces of graphene were picked up by PC/PDMS stamp and released on a substrate prepared in advance with bottom hBN and bottom graphite. The bottom graphite was designed to fully encompass the twisted bilayer graphene area, forming a global bottom gate.

### Electrical transport measurement

Electrical transport measurements were performed within an Oxford Instruments dilution refrigerator, maintaining a base phonon temperature of ∼10 mK. Keithley 2400 source-meters were used to apply bottom gate voltage. Stanford Research Systems SR860 lock-in amplifiers were employed to measure the four-terminal longitudinal resistance (*R*_xx_) and Hall resistance (*R*_yx_) using an AC current bias ranging from 0.1 to 10 nA at a frequency of 7.777 Hz, and the *R*_xx_ and *R*_yx_ were amplified utilizing a Stanford Research Systems SR560 voltage preamplifier.

### Twist angle extraction

To determine the twist angle between the top hBN and twisted bilayer graphene, we use the relationship *n*_s,GG_ = 8*θ*_GG_  ^2^/3^1/2^*a*^2^ at small twisted angle limit, where *θ*_GG_ represents the interlayer twist angle of twisted bilayer graphene, *n*_s,GG_ denotes the charge carrier density corresponding to the fully filled twisted bilayer graphene superlattice moiré unit cell, the value of *n*_s,GG_ is inferred from the *n*_s,GG_ = *C*_b_*V*_s,GG_/*e*, where *V*_s,GG_ represents the voltage value of the back gate corresponding to full filling peaks, *C*_b_ denotes the back gate capacitance, the values of *C*_b_ are determined by the

Landau fan diagram, and *a* = 0.246 nm signifies the interatomic distance in monolayer graphene. Then we convert the charge carrier density *n* to the twisted bilayer graphene moiré filling factor *v* (*v* = 4*n*/*n*_s,GG_) by utilizing a series of correlated insulating states corresponding to a series of distinct longitudinal resistance *R*_xx_ peaks, and we derive the twist angle in our device D1 as *θ*_GG_ = 1.15° and *θ*_GG_ = 1.09° for device D2.

To determine the twist angle of the top hBN twisted bilayer graphene, we use the relationship ${\lambda _{G/BN}} = \frac{{( {1 + \delta } )a}}{{\sqrt {2( {1 + \delta } )\,\,( {1 - {\mathrm{cos}}{\theta _{G/BN}}} ) + {\delta ^2}} }}$ and ${n_{s,G/BN}} = \frac{8}{{\sqrt 3 {\lambda _{G/BN}}^2}}$, where ${\theta _{G/BN}}$ represents the twist angle of top hBN and top graphene layer, *δ* represents the lattice mismatch between graphene and hBN, with a value of ∼1.7%, *n*_s,G/BN_ denotes the charge carrier density corresponding to the fully filled G/BN superlattice moiré unit cell. The method for determining the value of *n*_s,G/BN_ is the same as that for *n*_s,GG_. We attribute the additional superlattice peaks observed at 3 < | *v* | < 4 to the moiré superlattice formed between graphene and the top hBN, corresponding to four electrons or holes in the G/hBN moiré superlattice. These peaks are indicated by black vertical dashed lines in Fig. S8(e) and (f). This observation serves as evidence for the alignment with hBN, a criterion widely recognized and adopted in the research community [11,82]. We derive the twist angle between the top hBN and twisted bilayer graphene in our device D1 as *θ*_G/BN_ = 0.54° and *θ*_G/BN_ = 0.49° for device D2.

Theoretically, the appearance or absence of the zero-field Chern insulators in this system also depends on the commensurability between the graphene/graphene and graphene/hBN twist angles, which requires careful adjustment of the relative values of the two twist angles. The twist-angle-pairs in both of our devices are very close to the theoretically predicted commensurate configuration [83] which favors zero-field Chern insulators.

### Theoretical calculation model

We investigate the electronic properties of magic-angle twisted bilayer graphene (TBG) at integer fillings of the flat bands. We start with the famous Bistritzer–MacDonald continuum model [1] to depict the low energy effective Hamiltonian for TBG in the valley $\mu $ (for $K/K^{\prime}$ valley):


(1) \begin{eqnarray*}
H_\mu ^0 = \left[ {\begin{array}{@{}*{2}{c}@{}} { - \hbar {v_F}\!\Big( {{\bf{k}} - {\bf{K}}_1^\mu } \Big) \cdot {\sigma _\mu } + {V_{hBN}}}&{{U_\mu }\!\left( {\bf{r}} \right)}\\
{U_\mu ^\dagger\! \left( {\bf{r}} \right)}&\quad { - \hbar {v_F}\!\Big( {{\bf{k}} - {\bf{K}}_2^\mu } \Big) \cdot {\sigma _\mu }} \end{array}} \right],\ \ \ \ \ \
\end{eqnarray*}

where ${v_F}$ denotes the Fermi velocity, ${\bf{K}}_{1,2}^\mu $ represents two Dirac points from $\mu $ valley, and ${\sigma _\mu } = ( {\mu {\sigma _x},{\sigma _y}} )( {\mu = \pm } )$ are Pauli matrices in the sublattice space. The ${U_\mu }( {\bf{r}} )$ matrix describes the moiré potential between two graphene layers. The single aligned hBN substrate introduces an effective moiré potential term on the top layer of TBG [84]. In order to simplify our analysis, we assume that the moiré potential induced by the hBN and the moiré potential induced by the bilayer graphene share the same periodicity in real space. As a result, the moiré potential induce by hBN is given by:


(2) \begin{eqnarray*}
{V_{hBN}} &=& {V_0}
\left[ {\begin{array}{@{}*{2}{c}@{}} 1&\quad 0\\ 0&\quad 1 \end{array}} \right]
+ \Bigg\{ {V_1}{e^{i\mu \psi }}\Bigg[ \left[ {\begin{array}{@{}*{2}{c}@{}}
1&\quad {{\omega ^{ - \mu }}}\\ 1&\quad {{\omega ^{ - \mu }}} \end{array}} \right]{e^{i\mu {\bf{G}}_1^M \cdot {\bf{r}}}}\\
&&+ \left[ {\begin{array}{@{}*{2}{c}@{}} 1&\quad {{\omega ^\mu }}\\
{{\omega ^\mu }}&\quad {{\omega ^{ - \mu }}} \end{array}} \right] {e^{i\mu {\bf{G}}_2^M \cdot {\bf{r}}}} +
\left[ {\begin{array}{@{}*{2}{c}@{}} 1&\quad 1\\ {{\omega ^{ - \mu }}}&\quad {{\omega ^{ - \mu }}} \end{array}} \right] \Bigg]\\
&&\times \,{e^{ - i\mu \left( {{\bf{G}}_1^M + {\bf{G}}_2^M} \right) \cdot {\bf{r}}}} + h.c. \Bigg\}.
\end{eqnarray*}

where $\omega = {e^{i2\pi /3}}$, ${V_0} \approx $ 0.0289 eV, ${V_1} \approx $ 0.0210 eV and $\psi \approx $ −0.29 (rad).

Then we consider the dominant intravalley component of the long-range Coulomb interaction in the TBG system. The interaction Hamiltonian is given by:


(3) \begin{eqnarray*}
{H_C} = \frac{1}{{2{N_s}}}\mathop \sum \limits_{\lambda \lambda ^{\prime}}\ \mathop \sum \limits_{{\bf{kk^{\prime}q}}} \ V\left( {\bf{q}} \right)\hat c_{{\bf{k}} + {\bf{q}},\lambda }^\dagger \hat c_{{\bf{k^{\prime}}} - {\bf{q}},\lambda ^{\prime}}^\dagger {\hat c_{{\bf{k^{\prime}}},\lambda ^{\prime}}}{\hat c_{{\bf{k}},\lambda }}
\end{eqnarray*}

where ${N_s}$ is the number of moiré supercells in the system, and ${\bf{k}}$ and ${\bf{q}}$ are the wave vectors relative to the Dirac points; $\lambda \equiv ( {\mu ,\alpha ,\sigma } )$ represents the flavor, encompassing the valley $( \mu )$, layer-sublattice $( \alpha )$ points and spin $( \sigma )$ subspace. The Coulomb interaction can be described by: $V( {\bf{q}} ) = {e^2}{\mathrm{tanh}}( {| {\bf{q}} |{d_s}} )/( {2{\Omega _M}{\epsilon _{B{\mathrm{N}}}}{\epsilon _0}| {\bf{q}} |} )$ where ${\Omega _M}$ is the area of the moiré supercell, ${d_s} = $ 40 nm, ${\epsilon _{BN}} = $ 4 is the dielectric constant of hBN, and ${\epsilon _0}$ is the vacuum permittivity. We employ the Hartree–Fock approximation to the Coulomb interaction and self-consistently solve the Hamiltonian [85] ${H_0} + {H_C}$. We project the interaction Hamiltonian (with Hartree–Fock approximation) onto the flat band subspace. The remote bands below the charge neutrality point are all occupied, which can interact with the electrons in the flat bands. This effect can be described as a remote band potential acting on the flat band subspace [55,86], enhancing the band width of the flat bands to ∼50 meV and breaking the particle-hole symmetry. Furthermore, in addition to the screening effect from the metallic gate, the Coulomb interaction can be further screened by the virtual particle-hole excitation from the remote bands. We follow previous research [85] and characterize such screening effects using the constrained random phase approximation (cRPA).

We perform the unrestricted Hartree–Fock calculations within the low-energy flat band subspace including the remote band potential, with cRPA screened Coulomb interactions. The dominant order parameters in all the cases are ${\tau _z},{s_z}$ and ${\tau _z}{s_z}$, where $\tau $ and *s* are Pauli matrices defined in the valley and spin subspace, respectively.

Furthermore, we calculate the Wigner–Seitz radius *r*_s_ to characterize the formation of a Wigner crystal state. The Wigner–Seitz radius is defined as: ${r_s} = m_e^{\mathrm{*}}{r_e}/( {2\epsilon {m_0}{a_B}} )$ where ${r_e}$ is the average distance between the electrons in the moiré flat bands, which satisfies $\pi r_e^2 = 1/{n_e}$, and ${n_e}$ is the carrier density; $m_e^{\mathrm{*}}$ is the effective mass of electron and ${m_0}$ is the bare electron mass; ${a_B}$ is the Bohr radius.

### Symmetrize *R*_xx_ and anti-symmetrize *R*_yx_

Due to imperfect Hall geometry definition in our devices, there is a mixing of the *R*_xx_ and *R*_yx_, and we employed the standard procedure of symmetrizing and anti-symmetrizing the raw measured data to correct the mixing effect. This procedure allows us to obtain accurate values of *R*_xx_^S^ and *R*_yx_^AS^, respectively.

When performing measurements to obtain the Landau fan diagrams (fast sweeping axis: *ν*, slow sweeping axis: *B*), we employ the following symmetrize and anti-symmetrize procedure:


(4) \begin{eqnarray*}
R_{xx}^S\!\left( {{B_0},\nu } \right) &=& \frac{{{R_{xx,{\mathrm{raw}}}}\!\left( {{B_0},\nu } \right) + {R_{xx,{\mathrm{raw}}}}\!\left( { - {B_0},\nu } \right)}}{2},\\
R_{xx}^S\!\left( { - {B_0},\nu } \right) &=& R_{xx}^S\!\left( {{B_0},\nu } \right)\!;
\end{eqnarray*}

(5) \begin{eqnarray*}
R_{yx}^{AS}\!\left( {{B_0},\nu } \right) &=& \frac{{{R_{yx,{\mathrm{raw}}}}\!\left( {{B_0},\nu } \right) - {R_{yx,{\mathrm{raw}}}}\!\left( { - {B_0},\nu } \right)}}{2},\\
R_{yx}^{AS}\!\left( { - {B_0},\nu } \right) &=& - R_{yx}^{AS}\!\left( {{B_0},\nu } \right)\!.
\end{eqnarray*}

When performing measurements to obtain the hysteresis loops (with the sweeping axis as *B*, and *v* fixed at *v*_0_), we employ the following symmetrize and anti-symmetrize procedure:


(6) \begin{eqnarray*}
R_{xx}^ \uparrow\! \left( {B,{\nu _0}} \right) &=& \frac{{R_{xx,{\mathrm{raw}}}^ \uparrow\! \left( {B,{\nu _0}} \right) + R_{xx,{\mathrm{raw}}}^ \downarrow\! \left( { - B,{\nu _0}} \right)}}{2},\\
R_{xx}^ \downarrow\! \left( {B,{\nu _0}} \right) &=& \frac{{R_{xx,{\mathrm{raw}}}^ \downarrow\! \left( {B,{\nu _0}} \right) + R_{xx,{\mathrm{raw}}}^ \uparrow\! \left( { - B,{\nu _0}} \right)}}{2},\\
R_{xx}^ \uparrow \left( { - B,{\nu _0}} \right) &=& R_{xx}^ \downarrow\! \left( {B,{\nu _0}} \right)\!, \\
R_{xx}^ \downarrow\! \left( { - B,{\nu _0}} \right) &=& R_{xx}^ \uparrow \left( {B,{\nu _0}} \right)\!;
\end{eqnarray*}

(7) \begin{eqnarray*}
R_{yx}^ \uparrow\! \left( {B,{\nu _0}} \right) &=& \frac{{R_{yx,{\mathrm{raw}}}^ \uparrow\! \left( {B,{\nu _0}} \right) - R_{yx,{\mathrm{raw}}}^ \downarrow\! \left( { - B,{\nu _0}} \right)}}{2},\\
R_{yx}^ \downarrow\! \left( {B,{\nu _0}} \right) &=& \frac{{R_{yx,{\mathrm{raw}}}^ \downarrow\! \left( {B,{\nu _0}} \right) - R_{yx,{\mathrm{raw}}}^ \uparrow\! \left( { - B,{\nu _0}} \right)}}{2},\\
R_{yx}^ \uparrow\! \left( { - B,{\nu _0}} \right) &=& - R_{yx}^ \downarrow\! \left( {B,{\nu _0}} \right)\!, \\
R_{yx}^ \downarrow\! \left( { - B,{\nu _0}} \right) &=& - R_{yx}^ \uparrow \left( {B,{\nu _0}} \right)\!.
\end{eqnarray*}

## Supplementary Material

nwaf265_Supplemental_File
