# Supplementary material for: Cascade of zero-field Chern insulators in magic-angle bilayer graphene
Source: Natl Sci Rev. 2025 Jul 3;13(4):nwaf265. doi: 10.1093/nsr/nwaf265 (PMC12892360; doi:10.1093/nsr/nwaf265)
Supplement: nwaf265_Supplemental_File [file nwaf265_supplemental_file.pdf]

Supplemental Materials for

Cascade of Zero-field Chern Insulators in Magic-angle Bilayer Graphene

Zaizhe Zhang[1†], Jingxin Yang[1,2†], Bo Xie[3†], Zuo Feng[4], Shu Zhang[5], Kenji Watanabe[6], Takashi Taniguchi[7], Xiaoxia Yang[5], Qing Dai[5,8], Donghua Liu[9], Kaihui Liu[4], Zhida Song[1,10,11], Tao Liu[2*], Jianpeng Liu[3*] and Xiaobo Lu[1,10*]

[1]International Center for Quantum Materials, School of Physics, Peking University, Beijing 100871, China
[2]National Engineering Research Center of Electromagnetic Radiation Control Materials, University of Electronic Science and Technology of China, Chengdu 611731, China
[3]School of Physical Science and Technology, ShanghaiTech University, Shanghai 201210, China
[4]State Key Laboratory for Mesoscopic Physics, Frontiers Science Centre for Nano-optoelectronics, School of Physics, Peking University, Beijing 100871, China
[5]CAS Key Laboratory of Nanophotonic Materials and Devices, CAS Key Laboratory of Standardization and Measurement for Nanotechnology, CAS Center for Excellence in Nanoscience, National Center for Nanoscience and Technology, Beijing 100871, China
[6]Research Center for Electronic and Optical Materials, National Institute of Material Sciences, 1-1 Namiki, Tsukuba 305-0044, Japan
[7]Research Center for Materials Nanoarchitectonics, National Institute of Material Sciences, 1-1 Namiki, Tsukuba 305-0044, Japan
[8]School of Materials Science and Engineering, Shanghai Jiao Tong University, Shanghai, 200240, China
[9]School of Materials and Energy, University of Electronic Science and Technology of China, Chengdu 611731, China
[10]Collaborative Innovation Center of Quantum Matter, Beijing 100871, China
[11]Hefei National Laboratory, Hefei 230088, China

* Corresponding author. E-mails: liu.tao@uestc.edu.cn; liujp@shanghaitech.edu.cn; xiaobolu@pku.edu.cn
† Equally contributed to this work.

FIG. S1

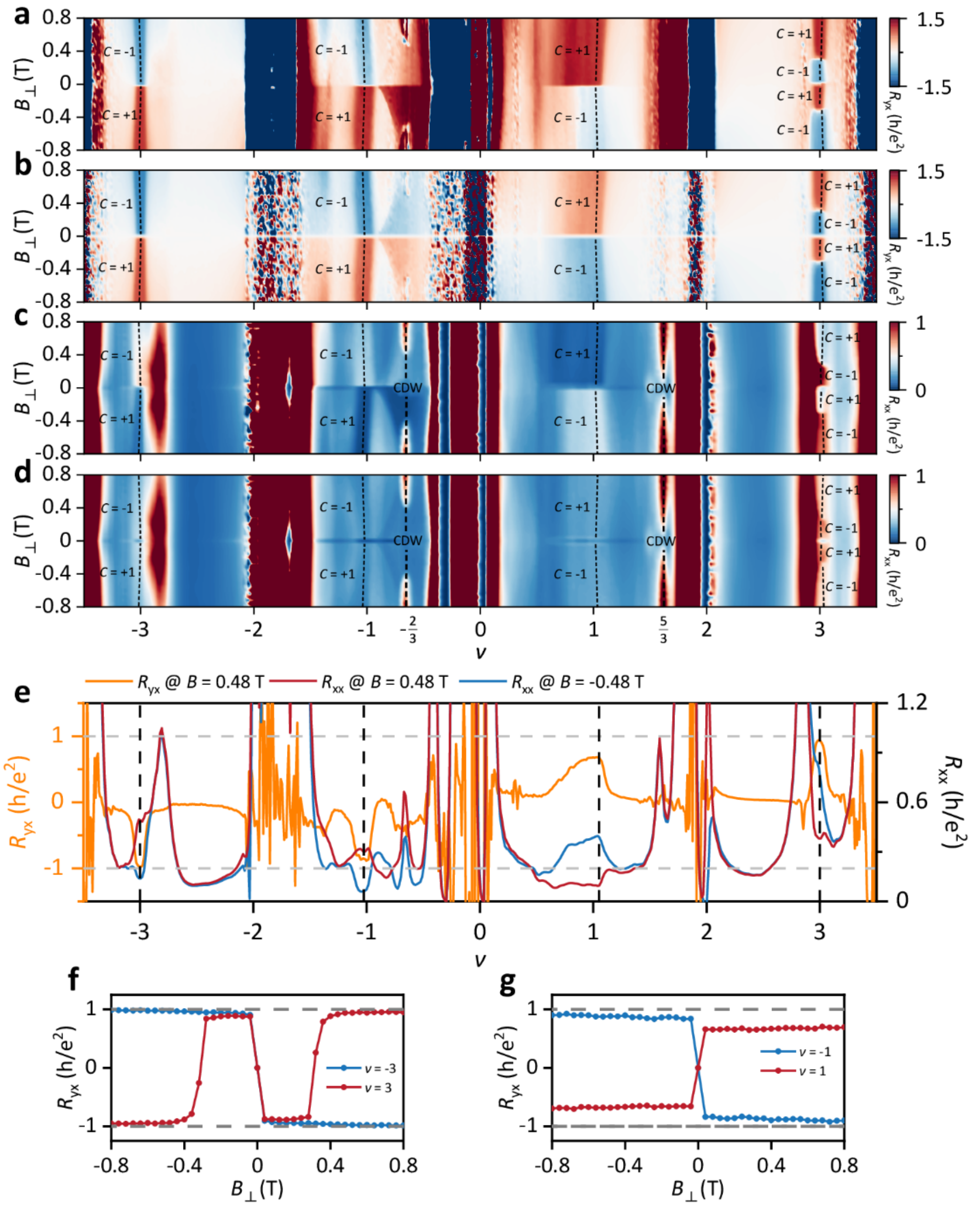

FIG. S1. Landau fan diagrams at low $B_\perp$ magnetic fields. (a) and (b) Unsymmetrized and anti-symmetrized R_{yx} versus filling factor v and out-of-plane magnetic field $B_\perp$ obtained at $T = 10$ mK. (c) and (d) Unsymmetrized and symmetrized R_{xx} versus v and $B_\perp$. The slanted dashed lines in the figure represent the evolution of the Chern insulator states with the magnetic field according to the Streda formula. Additionally, as the magnetic field increases, R_{xx} shows pronounced resistance peaks at filling factors $v = -2/3$ and $v = 5/3$, indicating the formation of emerging CDW states, as represented by vertical dashed lines in (c) and (d). (e) Line cuts of anti-symmetrized R_{yx} and unsymmetrized R_{xx} versus v at $B_\perp = \pm 0.48$ T. R_{xx} exhibits distinct dips at $v = 1$ and $v = 3$ when subjected to $B_\perp = +0.48$T, and similar dips are observed at $v = -1$ and $v = -3$ under $B_\perp = -0.48$T. Meanwhile, R_{yx} presents nearly quantized values at $v = \pm 3$ and $v = \pm 1$. (f) and (g) Anti-symmetrized R_{yx} versus out-of-plane magnetic field $B_\perp$ at $v = \pm 3$ and $v = \pm 1$ as extracted from Fig. 1(d).

FIG. S2

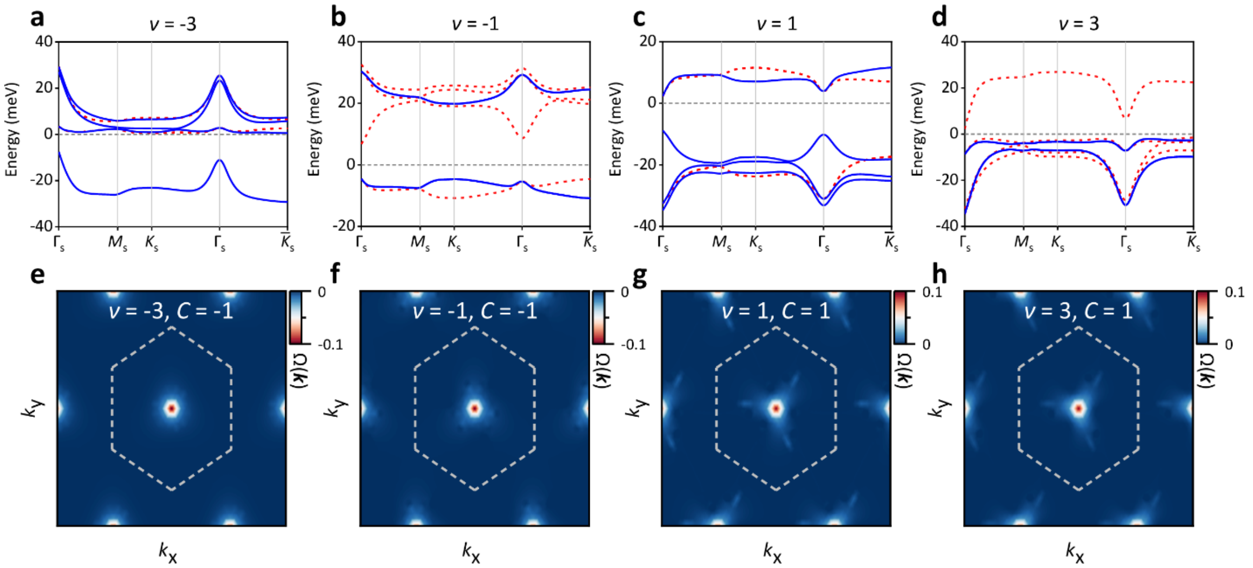

FIG. S2. Energy band, Berry curvature and Chern number at the $v = \pm 3$ and $v = \pm 1$ states obtained from theoretical calculations. (a)-(d) Band structure of the magic-angle TBG at the filling factor of v = -3, -1, 1 and 3. The red dashed lines and the blue lines denote the energy bands from two atomic valleys. (e)-(h) Distribution of the Berry curvature in the occupied flat band below the v = -3, -1, 1 and 3 gap. The gray dashed lines show the moiré Brillouin zone. The Chern number of the occupied bands is $C = +1$ for $v = +3$ and $v = +1$ under zero magnetic field and is $C = -1$ for v = -3 and v = -1.

FIG. S3

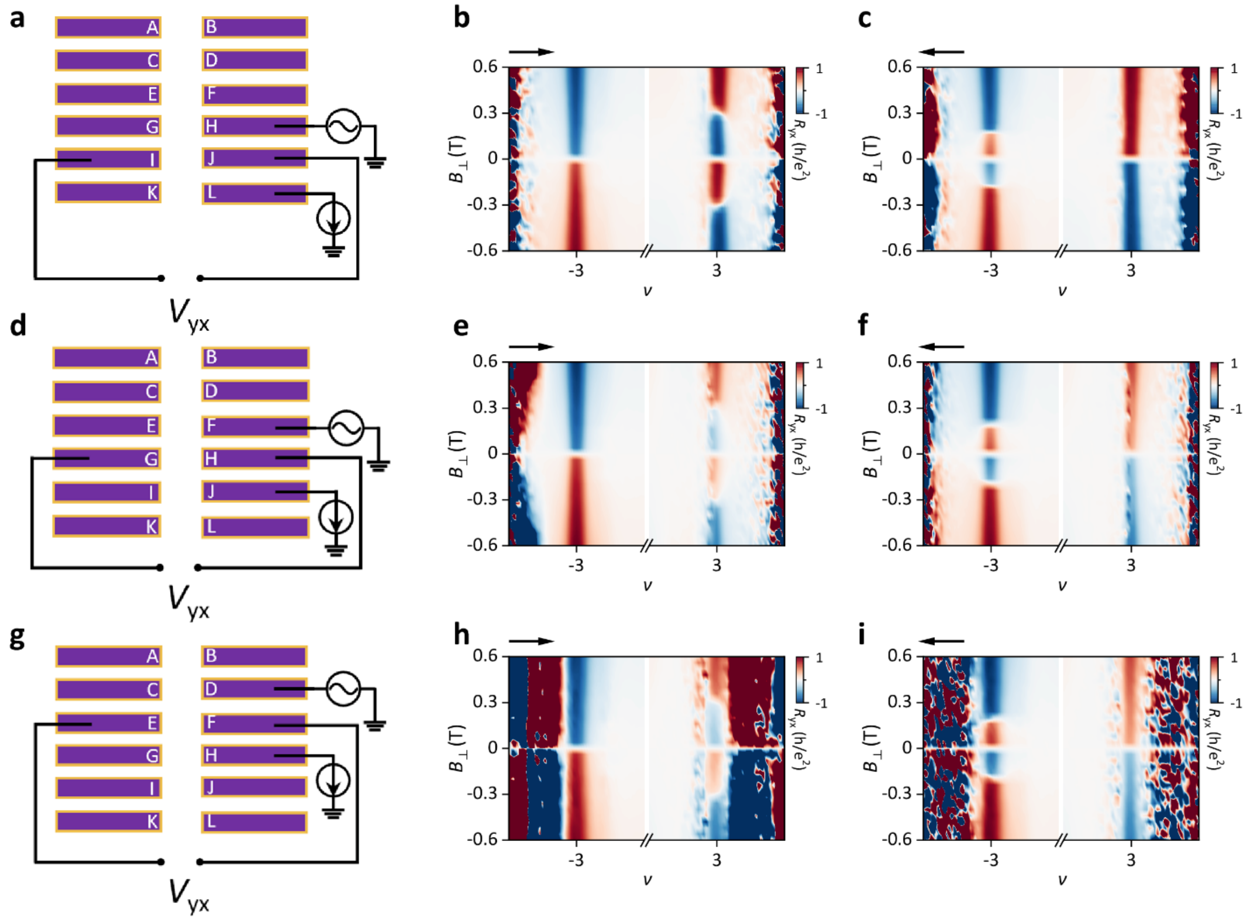

FIG. S3. Magnetic states determined by the gate sweeping direction. (a), (d) and (g) Measurement configurations for (b)-(c), (e)-(f) and (h)-(i) respectively. (b), (e) and (h) Anti-symmetrized R_{yx} versus filling factor v and out-of-plane magnetic field $B_\perp$ obtained at $T = 10$ mK when scanning the back gate from negative to positive voltage. (c), (f) and (i) Anti-symmetrized R_{yx} versus v and $B_\perp$ when scanning the back gate from positive to negative voltage. The arrows in the top left corner of the figure indicate the direction of the back gate scan. Upon scanning the back gate voltage from negative to positive, an additional sign reversal of R_{yx} is observed at the $v = 3$ state in the presence of a few hundred milli-Tesla magnetic field $B_\perp$. Conversely, when scanning from positive to negative voltage, the sign reversal occurs at the $v = -3$ state.

FIG. S4

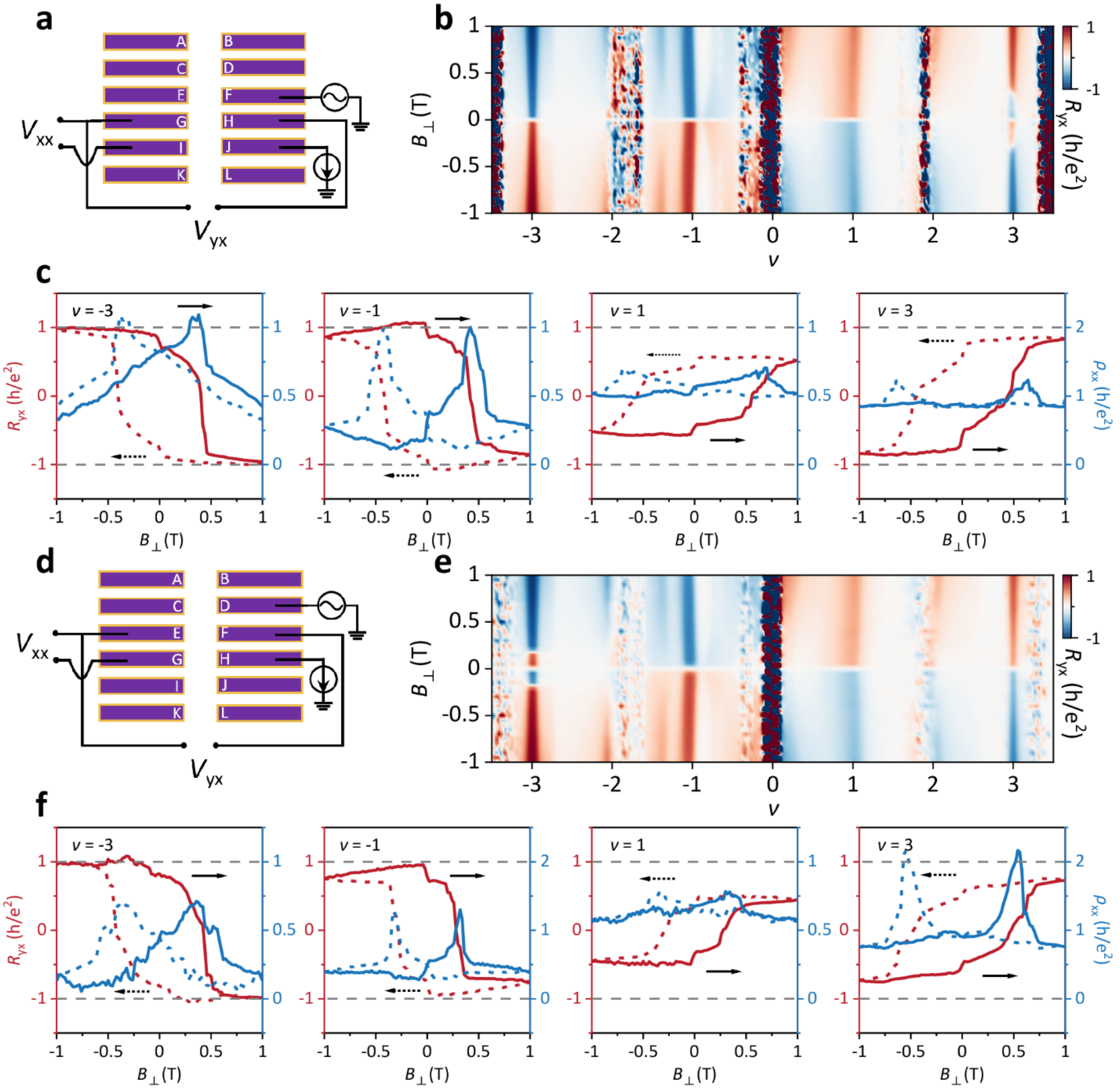

FIG. S4. Hysteresis loops and Landau fan diagrams of other Hall bar contact pairs in device D1. (a) and (d) Measurement configurations for (b)-(c) and (e)-(f) respectively. (b) and (e) Anti-symmetrized R_{yx} versus ν and $B_{\perp}$. (c) and (f) Symmetrized longitudinal resistivity ρ_{xx} and anti-symmetrized R_{yx} as a function of $B_{\perp}$ measured at $\nu = \pm 1$ and ± 3 states. Dashed and solid lines correspond to sweeping the $B_{\perp}$ field back and forth indicated by the arrows. All data are acquired at $T = 10$ mK.

FIG. S5

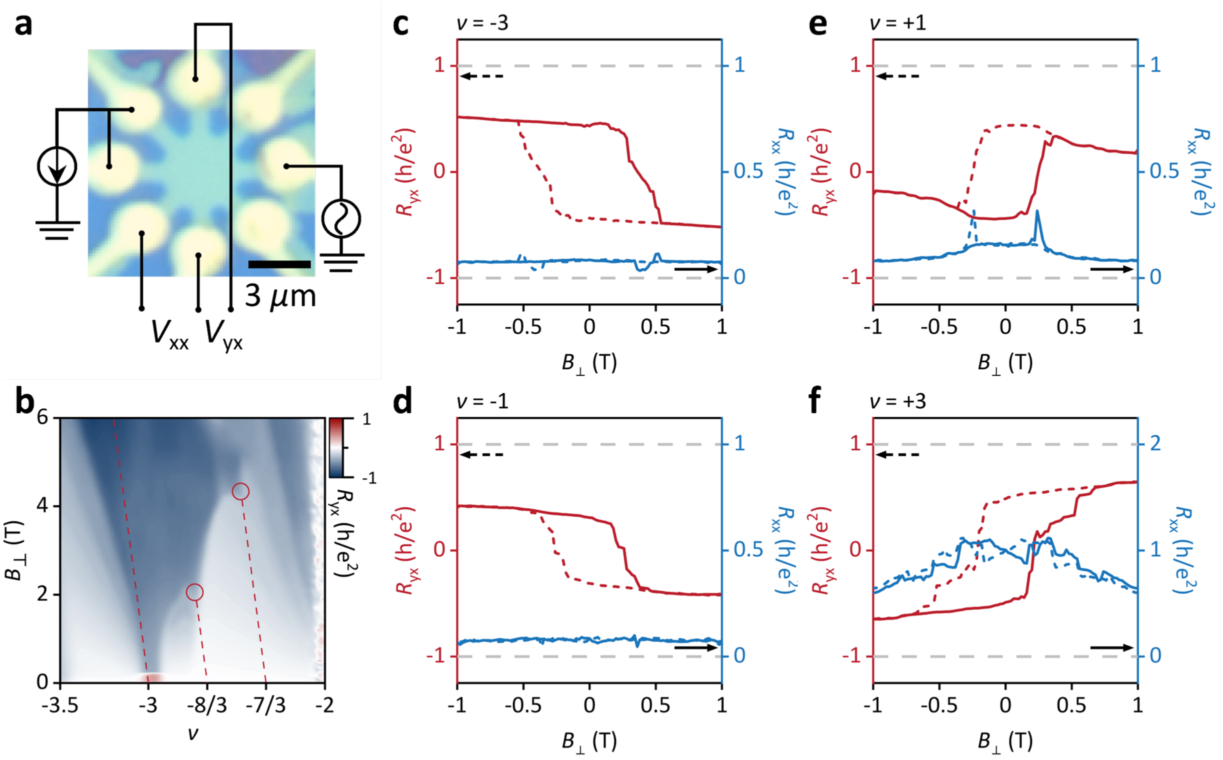

FIG. S5. Hysteresis loops and Landau fan diagrams of MATBG/hBN device D2 before reducing the sample area. (a) Optical microscopy image (scale bar is 3 μm) of device D2 and measurement configuration. (b) Anti-symmetrized R_{yx} versus ν and $B_\perp$. (c)-(f) Symmetrized longitudinal resistance R_{xx} and anti-symmetrized Hall resistance R_{yx} as a function of $B_\perp$ measured at $\nu = \pm 1$ and ± 3 states. Dashed and solid lines correspond to sweeping the $B_\perp$ field back and forth indicated by the arrows. All data are acquired at T = 10 mK. Which indicates the reproducibility of the commensurate and incommensurate Chern insulator states across different devices.

FIG. S6

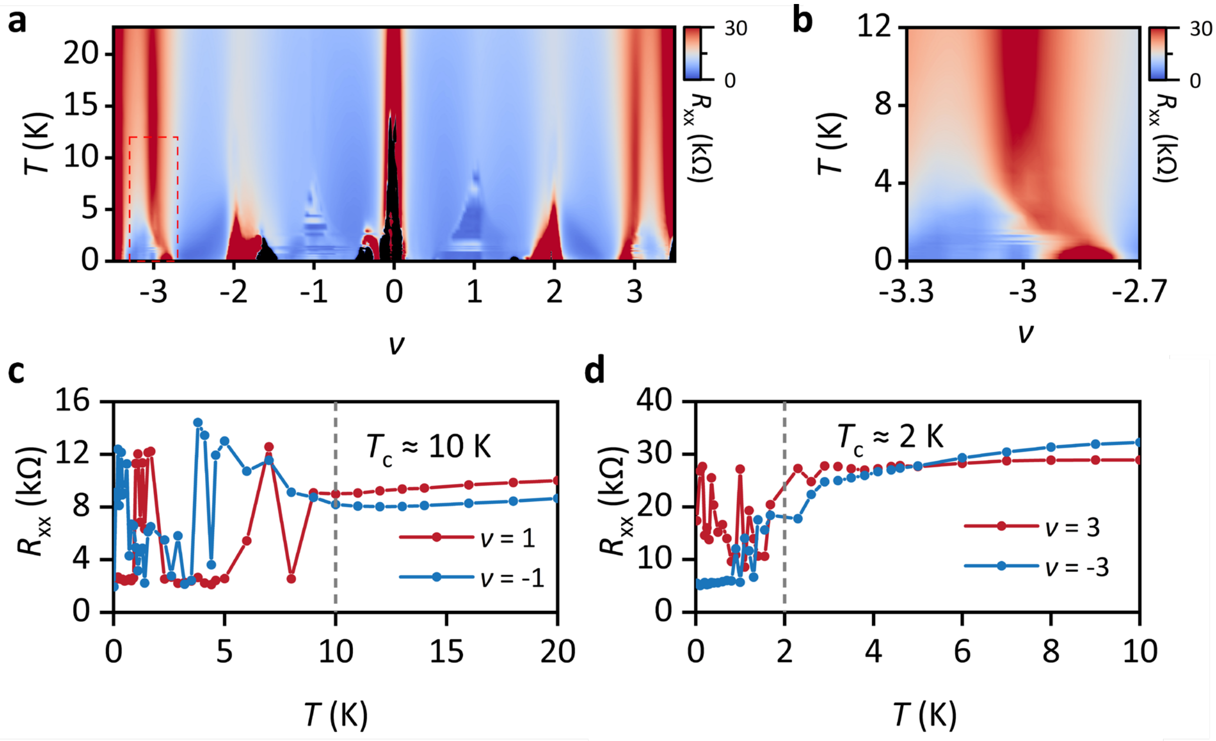

FIG. S6. Longitudinal resistance at different temperatures and filing factors. (a) Colormap R_{xx} (measured at zero magnetic field) against ν and T. (b) Zoomed-in R_{xx} colormap obtained from the region enclosed by the red dashed line in (a). (c) Linecut traces of R_{xx} versus T at $\nu = \pm 1$ from (a). (d) Linecut traces of R_{xx} versus T at $\nu = \pm 3$ from (a).

FIG. S7

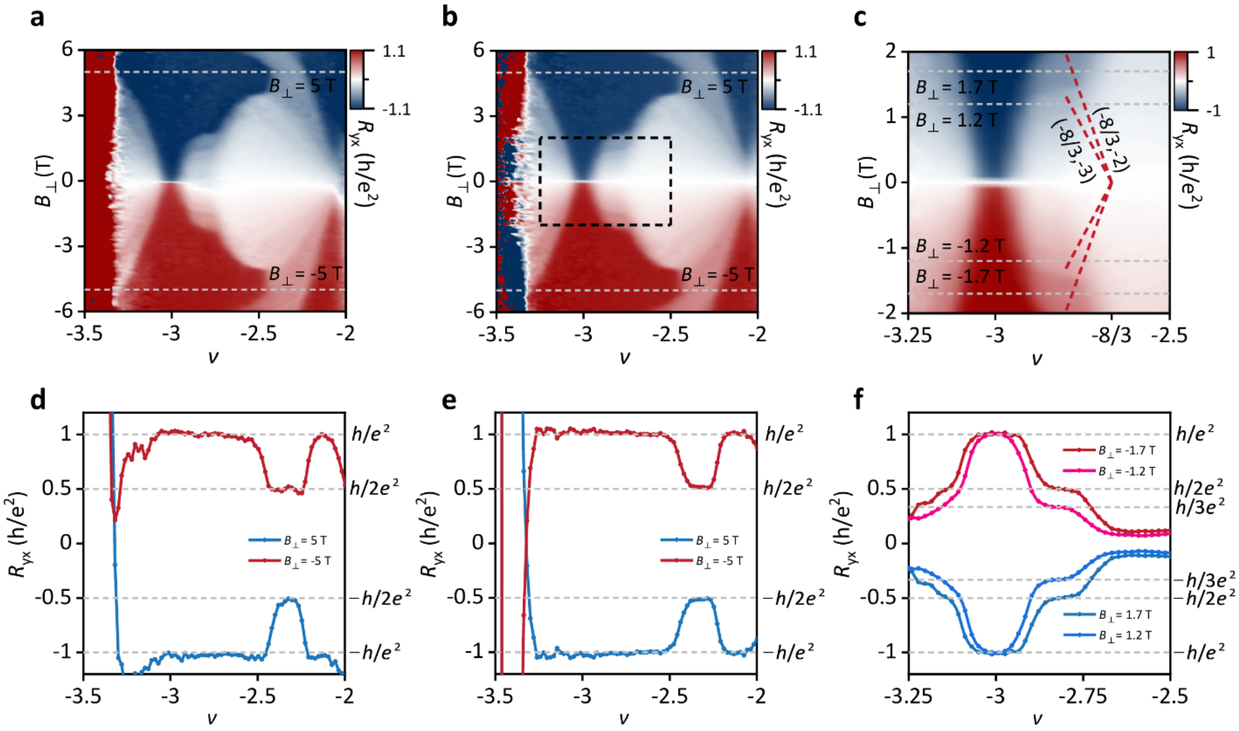

FIG. S7. Landau fan diagrams near v = -3. (a) and (b) Unsymmetrized and anti-symmetrized R_{yx} versus filling factor v and out-of-plane magnetic field $B_{\perp}$ obtained at T = 10 mK. (c) Anti-symmetrized R_{yx} map obtained in the boxed region in (b). The slanted dashed lines in the figure represent the evolution of the Chern insulator states (-8/3, -2) and (-8/3, -3) with the magnetic field according to the Streda formula. (d)-(f) Line cuts of R_{yx} corresponding to the gray dashed lines in (a)-(c) respectively.

FIG. S8

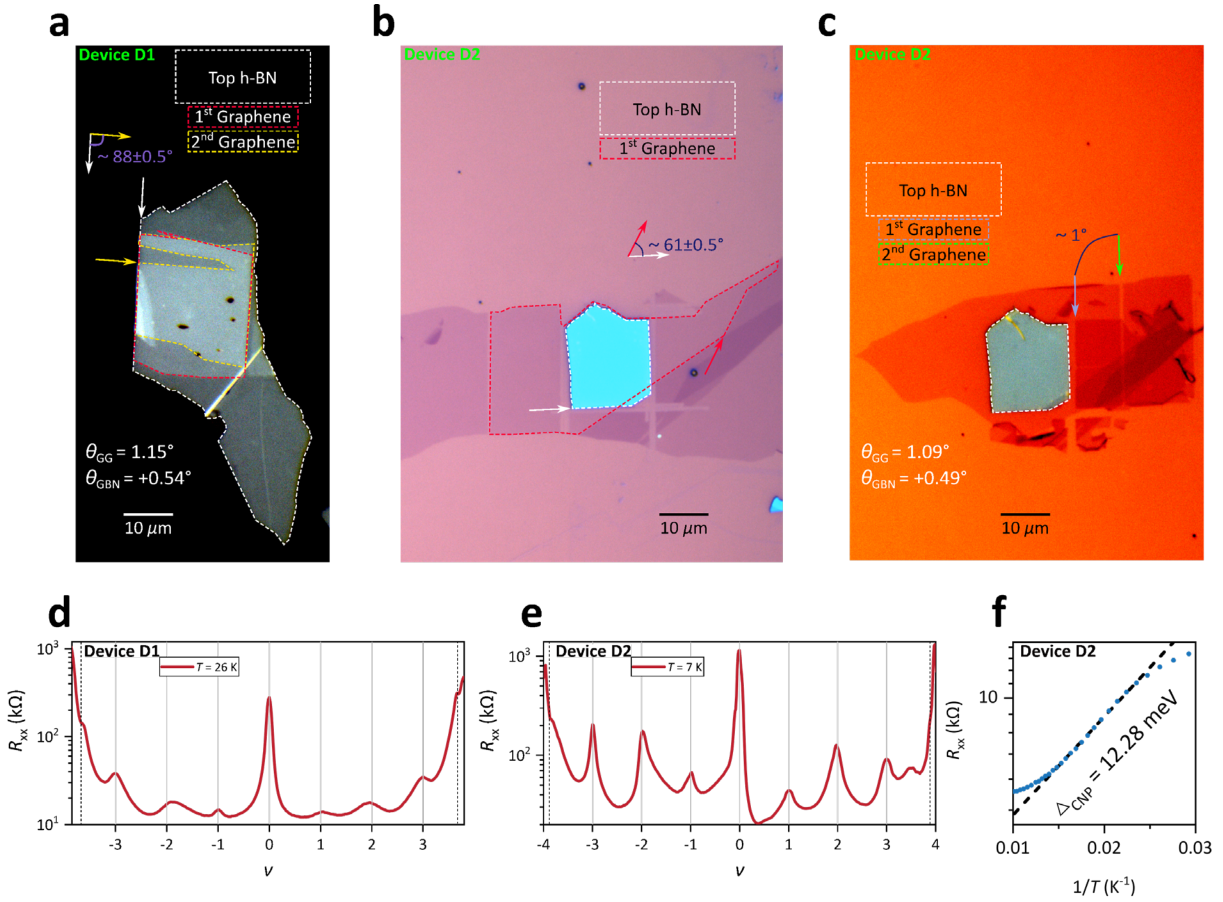

FIG. S8. Alignment between the twisted bilayer graphene and the top hBN in device D1 and D2. (a) Optical image obtained after using PC to pick up the top hBN and the two layers of graphene of device D1 during the stacking process, with white and yellow arrows indicating the crystalline orientations of the top hBN and the 2nd graphene layer, respectively. (b) Optical image obtained after using PPC to leave the top hBN on the 1st graphene and employing a laser to cut the 1st graphene along the boundary of the top hBN during the stacking process of device D2. The white and red arrows indicate the crystalline orientations of the top hBN and the first graphene layer, respectively. (c) Optical image obtained after using PC to place the top hBN/1st graphene onto the 2nd graphene during the stacking process of device D2. The green and blue arrows mark the laser-cut edges of the 1st and 2nd graphene layers, with an angular difference of approximately 1 deg, indicating a counterclockwise rotation of about 1 deg of the 2nd graphene layer relative to the 1st graphene layer. (d) R_{xx} as a function of TBG moiré filling factor ν at $T = 26$ K for device D1. (e) R_{xx} as a function of TBG moiré filling factor ν at $T = 7$ K for device D2. (f) Arrhenius plot for the $\nu = 0$ (CNP) in device D2, with an extracted gap of $\triangle_{CNP} = 12.28$ meV. The activation energy gap at the CNP is also very large, strongly suggesting a perfect alignment between the magic-angle twisted bilayer graphene and the hBN substrate.

FIG. S9

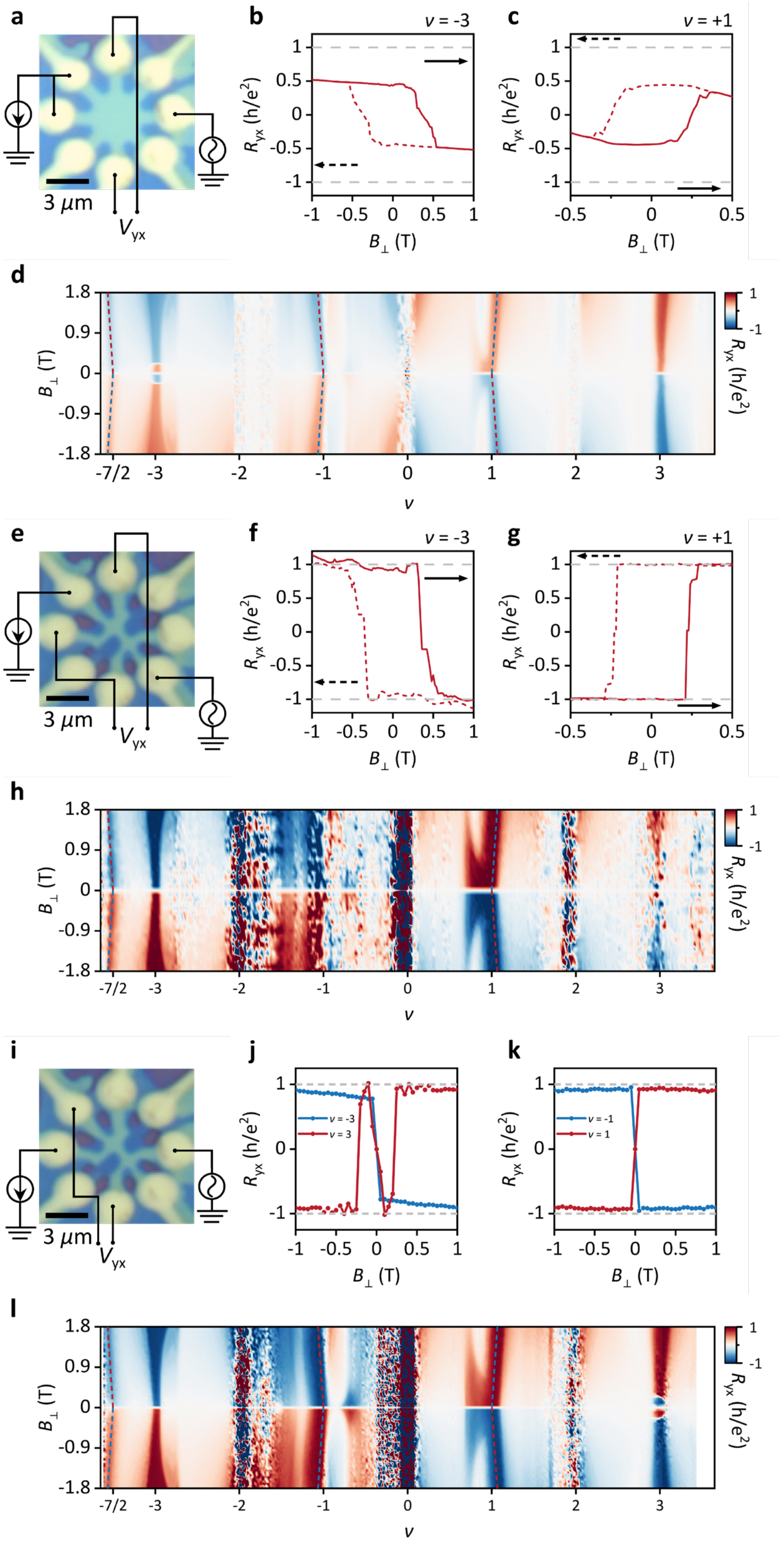

FIG. S9. Comparison of measurement results before and after reducing the sample area for device D2. (a), (e) and (i) Optical microscope images of device D2 before and after reducing the sample

area via etching and measurement configurations for (b)-(d), (f)-(h) and (j)-(l) respectively. (b)-(c) and (f)-(g) Hysteresis loops measured at v = -3 and +1 before and after reducing the sample area. Dashed and solid lines correspond to sweeping the out-of-plane magnetic field $B_{\perp}$ in opposite directions, as indicated by the arrows. (d), (h) and (l) Anti-symmetrized Hall resistance R_{yx} versus v and $B_{\perp}$, measured before and after reducing the sample area, the red dashed lines in the figure represent the evolution of the Chern insulator state (-7/2, -1), (-1, -1) and (1, 1) with the magnetic field according to the Streda formula. (j) and (k) Anti-symmetrized R_{yx} versus out-of-plane magnetic field $B_{\perp}$ at v = ±3 and v = ±1 as extracted from (l). All data are acquired at T = 10 mK. Which indicates that reducing the sample area significantly enhances both the anomalous Hall signal strength and the coercive field magnitude, thereby further improving the precision of quantization. Notably, at v = +1, the hysteresis loop shows that the anomalous Hall resistance at zero magnetic field improved dramatically—from reaching only 43.8% of the ideal quantized value before reducing the sample area to 99.8% afterward.
